# Supplementary material for: A realist interview study of a participatory public mental health project “#KindnessByPost”
Source: BMC Public Health. 2023 Dec 4;23:2406. doi: 10.1186/s12889-023-17372-2 (PMC10694983; doi:10.1186/s12889-023-17372-2)
Supplement: Supplementary file 1 — Supplementary Material 1 [file 12889_2023_17372_MOESM1_ESM.pdf]

Appendices

Appendix 1: Final list of CMOs (Context-Mechanism-Outcome statements)

Topic		If (context)	Then (outcome)	Because (mechanism)
Participation/ engagement	1.	If participants only need to commit to one contact with another person (i.e., sending one piece of mail to a person once)	then they may be more likely to participate	because there is no long-term commitment to growing a relationship/managing boundaries with a contact
	2.	If participants can choose which exchanges throughout the year, they sign up to	then they may be more likely to participate	because it feels more like a free choice to participate, rather than a chore that must be completed
	3.	If participants only see images of highly creative/high value mail on social media	then they may not want to participate	because they do not feel they can meet that perceived high standard
	4.	If potential participants do not have internet access	then they may not be able to participate independently	because they cannot register for the scheme
	5.	If potential participants do not use social media	then they may not hear about the scheme	because advertising typically happens thorough social media users sharing about #KindnessByPost
	6.	If potential participants have younger children	then they may participate for/with their children	because they want to model and encourage pro-social behaviour
	7.	If potential participants have little disposable income	then they may not be able to participate	because they need to pay for a stamp and craft/letter-writing materials
	8.	If potential participants listen to the #KindnessByPost podcast about creating mail	then they may be more likely to participate	because the content discussed discourages perfectionism and comparison against others, relieving that pressure in potential participants
	9.	If participants are recommended the scheme by somebody, they know personally	then they may be more likely to participate	because the scheme is endorsed by someone trusted
	10.	If there are nationwide events or circumstances that are negative	then people are more likely to participate	because they are seeking distraction/positivity/community to counteract feelings of negativity/isolation

	11.	If a participant is particularly passionate about kindness by post	then their social contacts may be more likely to participate	because they encourage others to participate through word-of-mouth or social media posts
	12.	If participants do not have much trust in their community/society	then they may be less likely to participate	because giving your name and address to a stranger requires you to trust that there will be a positive outcome
	13.	If participants only need to provide a first name and address for the exchange	then they may be more likely to participate	because the anonymity of this feels reassuring and limits potential negative outcomes
	14.	If there is a theme to the exchange	then participants may find it easier to create mail	because it gives participants a framework for what to write/make
	15.	If participants sign up because they want to spread kindness	then their benefit to wellbeing is primarily through sending their mail	because they value helping others over receiving something themselves
	16.	If participants sign up because they want to spread kindness	then they may still benefit from receiving mail	because you are allowing yourself to be vulnerable and receive kindness
	17.	If #KindnessByPost is supported by a good platform	participation is facilitated	because exchanges can be larger and are easier for founders to facilitate
	18.	If participants value #KindnessByPost	then they may post on social media about it and indirectly promote it	because they want the organisers and community to know the scheme is appreciated
	19.	If participants engage with website guidelines	then they may feel more able to participate	because the guidelines emphasise not needing to send something that is high value or highly creative
	20.	If participants are confident with English	then they may be more likely to participate	because there is not a language barrier in writing the card
	21.	If participants are female	then they may be more likely to participate	because being vulnerable and creative is societally a more feminine thing
	22.	If participants are female	then they may be more likely to participate	because the advertising/branding is more feminine
	23.	If exchanges are tied to a cultural event	then this does not make much difference to whether people sign up or not	because tying it to Christmas (or other cultural events) is not why people participate

	24.	If exchanges are tied to a cultural event	then participants may be more likely to participate	because they feel like cultural events may be times when people are more in need of connection
	25.	If exchanges are tied to a cultural event	then participants may be less likely to participate	because they do not celebrate that particular event
	26.	If #KindnessByPost advertising features homogenous people	then participants may be less likely to participate	because they may not think that the scheme is for someone like them
	27.	If a participant is the kind of person that likes to send cards or letters, or is of that generation where it was big	then they may be more likely to participate	because it is a familiar activity to them and something they value
	28.	If exchanges are every so often throughout the year	then people are more likely to participate	because it feels enough of a break from routine, rather than something done habitually every month
	29.	If a participant does not seem themselves as a creative person	then they may be less likely to participate	because they do not think they have the skills/standard required
	30.	If participant is anticipating the exchange	Then they feel ready for it	Because they have had time to think and prepare for the exchange
	31.	If participant participates in an exchange	it gives the participant a 'boost'	Because it reminds the participant that other people are kind
	32.	If a participant believes the scheme may help someone they know	They may encourage relatives (international) to join	Because they believe it will support or help them
	33.	If participant connects with the idea of #KindnessByPost (i.e., the ethos of it)	they are more likely to participate and wait for an exchange	Because the idea appeals to them regarding honesty of people, kindness, and card crafting (aligns with their values?)
	34.	If cards are received at times of the year when people may be particularly lonely	then the impact may be higher	Because the participant believes the card supports with feelings of connection when this is particularly needed
	35.	If participant finds participation meaningful to them	they are more likely to encourage others to sign up	Because they believe they exchange may support them too
	36.	If participant is a champion for the project	then they may recommend it to others	Because they believe the scheme to be fun and exciting, and want others to feel the way that they feel though participating

	37.	If participant likes the theme of the exchange	then they may be more likely to participate	because it emotionally connects with them
	38.	If participant experiences loneliness	then they may want to support others who are lonely through #KindnessByPost	because they feel a sense of empathy and want to help others who have been through what they have
	39.	If participant takes part in the exchange	they will feel uplifted	because something lovely has happened to them
	40.	If participants have a good experience of engaging in the scheme where it supports them	they are more likely to endorse the scheme and recommend it to others	because they believe it could support their mental health
	41.	If participants have participated in similar schemes	they may be more likely to participate	because they may have had previous nice experiences and may also confuse the schemes
	42.	If participant is going through a difficult time	then the participant may feel attracted to the scheme	because there are no expectations and its completely random
	43.	If participant has previous experiences of strangers improving their mental health	they may be more likely to participate	because they understand that it is possible for a stranger to brighten someone's day
	44.	If someone hears about kindness by post	they may be more likely to sign up to other kindness schemes	because they feel inspired by #KindnessByPost
	45.	If participant takes part in kindness by post	it may provide a belief that there are good people out there especially if you are going through a difficult time	because it is a pick-me-up that somebody case when you are alone
	46.	If someone participates in kindness by post	they may engage in similar acts of kindness elsewhere in their lives	because it is the kind of way, they tend to show kindness outside of the scheme
	47.	If participants are time-poor	they may participate	because they feel the commitment is quite small
	48.	If participants are time-poor	they may not participate	because they do not have the capacity to take the project on
	49.	If participants engage in the scheme	they may recommend it to others	because they believe it is a lovely thing for people to do

	50.	If participants engage in the scheme	they may feel a sense of connection with others	because of the sense of making life easier for someone else
	51.	If participants engage in the scheme	it may help to give them a lift even if they have people around them	because it affects them positively and emotionally
	52.	If participants engage in the scheme	it may provide a sense of hope	because it helps you believe that life is going to get better
	53.	If exchanges as connected to too many special days	it could start discriminating against people	because the exchanges will not feel like they are for everyone
	54.	If participant has previous experiences of sending and receiving mail to strangers	then they are more likely to see the benefits of participating	because they like the idea and have a sense of how it may help
	55.	If participant heard about the exchange in a magazine that they read	then they may be more likely to sign up	because they are reading about it somewhere which aligns with their own interests
	56.	If participant reads about #KindnessByPost in their magazines	they may feel that #KindnessByPost is for educated and wealthier people	because they consider these to be the kinds of groups who read their kinds of magazines
	57.	If #KindnessByPost created partnerships with charities and health organisations	then it may help to reduce barriers of access	because they could target groups who are not currently participating
	58.	if the participant is naturally good at talking to strangers	then people are more likely to participate	because they find it easier to write a card to someone
	59.	If the Mental Health Collective email or send calendar reminders about #KindnessByPost to all their members	then people are more likely to participate	because they are given a prompt from a trusted source
	60.	If participants know #KindnessByPost is set up as a collective card exchange	then they may be more likely to participate	because they know the other person wants a card and their act of kindness is likely to be well received
	61.	If the #KindnessByPost registration and admin processes are quick and simple	then people may be more likely to participate	because it is not arduous and time consuming

	62.	If there are not many other mutual kindness schemes around	then #KindnessByPost will be particularly attractive to take part in	Because it is offering something different, which people cannot find elsewhere
Giving mail	63.	If participants see images of different types of mail posted on social media	then they may find it easier to create mail	because others' creations provide inspiration and ideas for their own creations
	64.	If it is a participants' first time doing an exchange	then they may find it difficult to create mail	because they may be unsure about what to write to a stranger
	65	If participants do not know anything about the person, they are sending mail to	then creating mail may be particularly effective in boosting mood	because creating mail becomes an introspective exercise, in which participants create things or write about things that bring them joy
	66.	If participants do not know anything about the person, they are sending mail to	then they may find it easier to create mail	because if they had some limited information about their recipients' personality/preferences, this could add complex parameters around what they felt they should write or make to suit them
	67.	If participants do not know anything about the person, they are sending mail to	then they may find it difficult to create mail	because they do not know what the recipient would like or what messages they need to hear
	68.	If participants do not know anything about the person, they are sending mail to	then creating mail may be particularly effective in boosting mood	because the anonymity of the exchange/knowing there will not be a response allows a participant to be less guarded and express their thoughts/feelings/fears more freely
	69.	If participants create their mail themselves	then this is an enjoyable experience	because it is a chance for self-expression
	70.	If participants create their mail themselves	then this is an empowering/confidence-building experience	because there is a sense of achievement at having done something to benefit somebody else
	71.	If participants create their mail as a group activity with others	then this may be a bonding experience for the group	because the group is united in a common task to do a kind thing for a stranger

72.	If participants home-make a very simple piece of mail	this may make it easier for others to participate	because they demonstrate that mail does not need to be outstanding and lower the bar for everyone
73.	If participants do not know anything about the person, they are sending mail to	then they may quote things are write about more general stuff	because they want it to be positive for the recipient regardless of who they are
74.	If participants create their mail themselves	then this is an enjoyable experience	because it is a chance to engage in an enjoyable activity you might not otherwise get to do
75.	If participants create their mail themselves	then this is an enjoyable experience	because it is a chance to be creative
76.	If participant creates the card themselves	they feel they help themselves and are excited	Because they challenge oneself and have a nice feeling
77.	If participant reflects on the importance of human-to-human connection and values this	they are more likely to believe in the importance of #KindnessByPost	Because their values align with that of the scheme
78.	If participant decides to make their own card	it increases creativity	Because they find simple creative solutions online like on YouTube
79.	If participant tries something creatively new	it increases a sense of learning and accomplishment	Because they have overcome challenges, they did not think were possible to overcome
80.	If participant believes it is important to send the 'right' card	they may spend time thinking about what to write/create	Because they are sensitive to what others may be going through
81.	If participant is inspired by others to participate	they may be more likely to engage in creative activities	Because they purchase craft materials to create a card
82.	If participant needs to support to engage and receives it	they may feel that the exchange is accessible	Because they are still able to participate despite barriers such as online access
83.	If participant has other commitments and limited time	they may be more likely to participate in the scheme	Because the commitment to participate is small and you can do it in your own time
84.	If participant writes a letter to go with their card	they may feel that it is a therapeutic process	because they have had an opportunity to engage emotionally in the process
85.	If participant makes a card	they may feel a sense of autonomy and be able to check in with their feelings	because they do not feel restricted by the exchange theme

	86.	If participant goes away on holiday	they may find inspiration for making and sending cards	because they have time to reflect
	87.	If participant writes to a stranger	they use less emotive grammar	because it does not feel appropriate to share too much
	88.	If participant buys a card	they may write inspirational motivational quotes or write content that fits the theme	because they want to send something meaningful
	89.	If participant puts thought into what they write on their card	they may find it to be a creative process	because they are reflecting and engaging in the process
	90.	If participant has had emotional responses to words in the past	they may be more thoughtful in their creations	because they are aware of how powerful the written word can be
	91.	if participants do not feel very creative or do not have time to make a card	then they are more likely to buy a card	because the creative aspect of sending a card is challenging for them
	92.	If participants are very perfectionist and anxious by nature	then making and giving a card may be more stressful and less enjoyable	because they will worry about whether their card is good enough
Receiving mail	93.	If received mail is personalised	then the recipient's wellbeing may more improved	because they appreciate the time/effort that the sender put into doing something kind for a stranger, over mail that feels rushed or low effort
	94.	If participants do not receive mail in the exchange	then they may not be negatively impacted	because the knowledge that kindness heroes exist is enough of a benefit as they serve as a demonstration of selfless kindness
	95.	If participants do not receive mail in the exchange	then they may be negatively impacted	because they would like a card, but do not want to request one as they do not want to create extra work for another participant
	96.	If participants do not receive mail in the exchange	then they may not be negatively impacted	because they do not feel that they need one to get value from the exchange, as often participants get most reward from creating mail
	97.	If participants do not receive mail in the exchange	then their wellbeing may be negatively impacted	because they do not have enough self-esteem to feel as though they can request a card

	98.	If mail is received in the exchange	then it can protect positive wellbeing	because the positive experience of a stranger taking time to spread kindness counteracts perceived negative actions carried out by other people
	99.	If participants do receive mail in the exchange	the it may be a boost rather than the main value drawn from the project	because the participant mostly signed up to spread kindness to others
	100.	If mail is received in the exchange	then it can boost wellbeing	because it is a positive experience to receive kindness from a stranger
	101.	If mail is received in the exchange	participants may not share it online	because they see it as something personal to them and do not want to violate sender's privacy
	102.	If participant receives a card	they may use this as inspiration for their own card creation in the future	Because they like the messages they received and the way it made them feel
	103.	If participant does not receive a card	they may feel like they are waiting for it to arrive, expectations are not met and there is a sense of being left down	Because they have been told they may receive a card
	104.	If participant receives a card	it may reduce stress	because it breaks up the routine of working from home
	105.	If a participant receives any kind of mail in the exchange	then the recipient's wellbeing may more improved	because it is an unusual experience that stands out from other mail/disrupts routine
	106.	If participant receives a card from a child	they may feel the scheme is not discriminating age-wise	because they love to receive post from children
	107.	If participant does not receive a card	they may feel left out	because they expected to receive a card
	108.	If participant is expecting/ waiting for a card	then they may have a shared experience with their family	because they all wait together for it to arrive
	109.	If #KindnessByPost is not set up as a mutual exchange and you receive mail	then the positive impact on wellbeing may be bigger/more certain	because you do not make invidious comparisons between the card you gave and the card you got

		from someone different from who you sent a card to		
Content of received mail	110.	If participants see value of received cards primarily as the effort made by a stranger	then it boosts wellbeing regardless of whether it is a letter/gift/handmade card	because any handwritten/handmade piece of mail feels effortful and personal
	111.	If a participant receives any kind of mail in the exchange	then participants feel they identify with the person they share the exchange with	because it is a tangible reminder of the kindness of others
	112.	If what is written in received mail is applicable to current circumstances	then this can feel particularly meaningful/helpful to wellbeing	because it can feel like it was the right message at the right time; a deeper connection with the mail and the person who wrote it
	113.	If the sender has made assumptions about the beliefs/personality of the recipient	then this can detract from the joy of receiving mail	because the recipient feels alienated if judgements made about them are incorrect
	114.	If received mail is personalised or a lot of effort	then the recipient's wellbeing may more improved	because they appreciate the time/effort that the sender put into doing something kind for a stranger, over mail that feels rushed or low effort
	115.	if you do not know anything about the person you are exchanging with	then you can get still get a lot of value from receiving your card	because just being kind humans together is enough of a connection
	116.	If a participant receives any kind of mail in the exchange	then the recipient's wellbeing may be improved	because kindness from a stranger is more powerful than kindness from somebody you know, because there is no transactional relationship
	117.	If a participant receives any kind of mail in the exchange	then the recipient's wellbeing may be improved	because it is something that feels more effortful/special than an email or text
	118.	If participant receives a gift in the exchange	this may not be beneficial to their wellbeing	because it does not feel in line with the aim of the scheme

	119.	If participant chooses to write their contact details on the card	they may enter into a longer-term exchange with a stranger and create connections	because they are open to removing anonymity and sharing more about themselves
	120.	If participant puts thought into what they write on their card	they may still upset the person who receives it	because different people are reminded of different things through language, even if unintentional
	121.	If there is something about the card that inspires them	then it can encourage them to take part in future exchanges	because they look forward to sending the card and the impact it will have
Long-term	122.	If received mail is kept and displayed prominently in the home	then this may improve mood	because it acts as a day-to-day physical/visual reminder of the kindness of others/the #KindnessByPost community
	123.	If participants plan to participate in future exchanges	then they may have a sustained improvement in wellbeing	because outside of exchange periods they are primed to take note of positive messages that could go in future mail
	124.	if participants keep mail	then they may have a sustained improvement in wellbeing	because they can re-read and appreciate the kindness many times
	125.	if participants keep mail	then others may benefit from them too	because they share their cards with friends and family
	126.	If gaps between exchanges are made shorter	motivation and wellbeing benefits increase	Because it gives strength to carry on and you feel part of a community
	127.	If the participant continues to try something creatively new	it builds confidence	Because the participant feels better about themselves and learns to take more creative risks
	128.	If participant participates regularly and looks forward to the next exchange	then it may boost positivity	because they know what to expect of the exchange
	129.	If participant engages in #KindnessByPost long-term	they may feel the themes of the exchange are less important	because it is the regularity of it that they consider important

	130.	If Kindness by post inspires them to send letters to friends and family	then they may have a sustained improvement in wellbeing	because they get positive benefits from sending and receiving cards to family and friends
	131.	If the person takes part in the scheme	then this may have long-term benefits for wellbeing	because it promotes a mindset of doing kindness and seeing the world as a kind place, which goes beyond the exchange itself
	132.	If someone takes part in #KindnessByPost exchanges regularly	then it can have a sustained benefit for wellbeing	because there is constantly some kindness in the person's life/mind
	133.	If participant receives other postcards and letters from friends outside of the exchange	then they are more likely to display their #KindnessByPost cards alongside them	because it is a cultural practice for them to display cards in the home
Community	134.	If made/received mail is shared on social media	then a sense of community is reinforced	because participants are reminded that there are more than just themselves/their sender/their recipient participating
	135.	If participants talk about where they live or talks about where the recipient lives	then a sense of community may be developed	because participants learn about where their recipient lives, or where their sender lives through the exchange
	136.	If participants post about their exchange on social media	then they are more likely to post about the mail that they created	because it is less vulnerable to post something you have made
	137.	If participants post mail on social media	then they may be seeking additional connection	because they want people to see their mail has been received, or let them know they were the person that got that one
	138.	If participant engaged in scheme with colleagues	A sense of community is created	Because there is a sense of shared experience and everyone doing something new together
	139.	If participant shares exchange experiences with family	It provides something to talk about and a sense of being like-minded	Because families share the kind words and fun experiences connected to their exchange (re: receiving card)
	140.	If participant shares exchange experiences with family	it brings joy to them to bring joy to someone else	because they see them happy, and it is nice to see someone else smile

	141.	If participant feels the words of the card are meaningful	it increases confidence and hope in other people	Because they feel they are part of something and part of a shared community
	142.	If participant sees others benefiting from #KindnessByPost	then they may feel benefits themselves	because they feel it is a lovely thing to be a part of
	143.	If participant sees positive messages on social media	then they may feel more positive and hopeful about the world	because it counteracts the usual negativity that they see online
	144.	If the participant takes part in random acts of kindness exchanges	then a sense of community may be developed	because participants feel closer to their community - stronger sense of community
	145.	If participant sends of receives their card to/from someone from a different ethnic background or cultural community	their mental health may benefit more	because they learn about different communities
